# A Benchmark Environment for Neuromorphic Stereo Vision

**DOI:** 10.3389/frobt.2021.647634

**Published:** 2021-05-19

**Authors:** L. Steffen, M. Elfgen, S. Ulbrich, A. Roennau, R. Dillmann

**Affiliations:** Interactive Diagnosis and Service Systems (IDS), Intelligent Systems and Production Engineering (ISPE), FZI Research Center for Information Technology, Karlsruhe, Germany

**Keywords:** 3D reconstruction, benchmark, event-based stereo vision, neuromorphic applications, neuromorphic sensors

## Abstract

Without neuromorphic hardware, artificial stereo vision suffers from high resource demands and processing times impeding real-time capability. This is mainly caused by high frame rates, a quality feature for conventional cameras, generating large amounts of redundant data. Neuromorphic visual sensors generate less redundant and more relevant data solving the issue of over- and undersampling at the same time. However, they require a rethinking of processing as established techniques in conventional stereo vision do not exploit the potential of their event-based operation principle. Many alternatives have been recently proposed which have yet to be evaluated on a common data basis. We propose a benchmark environment offering the methods and tools to compare different algorithms for depth reconstruction from two event-based sensors. To this end, an experimental setup consisting of two event-based and one depth sensor as well as a framework enabling synchronized, calibrated data recording is presented. Furthermore, we define metrics enabling a meaningful comparison of the examined algorithms, covering aspects such as performance, precision and applicability. To evaluate the benchmark, a stereo matching algorithm was implemented as a testing candidate and multiple experiments with different settings and camera parameters have been carried out. This work is a foundation for a robust and flexible evaluation of the multitude of new techniques for event-based stereo vision, allowing a meaningful comparison.

## Introduction

Frame-based visual sensors produce large amounts of redundant data while operating with a limited temporal resolution, which leads to over and undersampling at the same time. Meaning huge amount of redundant data with an insufficient resolution is generated. Event cameras, due to their asynchronous operation principle, overcome both issues opening up new opportunities for computer vision Delbruck (2008) and Delbruck (2016). As artificial stereo vision has always been especially suffering from high resource demands and computation times, researchers developed many event-based techniques for this problem Steffen et al. (2019). Beside profiting from less redundant data, event-based methods offer an additional matching criterion: Time. An important assistance to develop sophisticated algorithms and improve the state-of-the-art in any field are standardized comprehensive evaluations. A profound and reasonable evaluation of any algorithm greatly benefits from open benchmark datasets. While they have been done intensively for frame-based stereo vision Seitz et al. (2006); Scharstein and Szeliski (2002), comparable studies are still missing for respective event-based techniques.

A comprehensive overview of the biological and technical background of neuromorphic visual sensors is given in Posch et al. (2014); Delbruck (2016); Steffen et al. (2019), including a comparison of well known exponents like the Dynamic Vision Sensor (DVS), the Asynchronous Time-based Image Sensor (ATIS) and the Dynamic and Active Pixel Vision Sensor (DAVIS). A survey on event-based vision focusing on applications and algorithms was done in Gallego et al. (2019). Furthermore, Steffen et al. (2019) provides a survey of event-driven depth perception. Depth reconstruction from event streams can be categorized in spiking and non-spiking approaches; the former does apply Spiking Neural Networks (SNN) and the latter does not. Examples for spiking stereo vision approaches are techniques like Dikov et al. (2017); Kaiser et al. (2018); Osswald et al. (2017) which are based on the cooperative algorithm Marr and Poggio (1976) and its extensions with Gabor filters Camunas-Mesa et al. (2018) or Belief Propagation Xie et al. (2017). An interesting example for an SNN-based approach working with an entirely different technique is demonstrated in Haessig et al. (2019). Here the depth information is correlated to the amount of defocus present at the focal plane. This approach works with only one sensor using a focus-tuneable lens. To meet the demand of open benchmarks, the large event-based vision datasets, like Li et al. (2017) focusing on pattern recognition and object classification and Hu et al. (2016) emphasizing dynamic vision tasks, were published. A review about benchmarks in event-based vision is provided in Tan et al. (2015) and in the scope of Frontiers’ research topic *Benchmarks and Challenges for Neuromorphic Engineering*, many researchers published standard datasets recorded with neuromorphic vision sensors. This includes Barranco et al. (2016) targeting navigation, Rueckauer and Delbruck (2016) regarding optical flow as well as Liu et al. (2016), in which DVS recordings are complemented by simulated event-based data generated from images with spike encoding. The vast majority of datasets and benchmarks for event-based data is done with the DVS Liu et al. (2016); Li et al. (2017) or the DAVIS Barranco et al. (2016); Rueckauer and Delbruck (2016); Hu et al. (2016). In contrast to that, we use the ATIS. Furthermore, many benchmark datasets regarding event-based vision, like Barranco et al. (2016); Rueckauer and Delbruck (2016); Liu et al. (2016); Li et al. (2017), are recorded with only one sensor. In our benchmark environment, a stereo setup allowing 3D reconstruction is applied. The objective of this work is to develop a benchmark environment providing the methods and tools to evaluate algorithms for event-based depth reconstruction. This allows a comprehensive and flexible evaluation for respective algorithms in a reproducible fashion. To this end, we designed and implemented a setup capable of capturing calibrated and synchronized data from event-based sensors while providing a ground truth for the depth information. To compare different approaches in a meaningful and reproducible way, metrics are defined covering core aspects like performance, precision and applicability of the examined algorithm.

## The Benchmark Environment

The theoretical concept of our benchmark environment consists of three parts. Data acquisition, sensor calibration and evaluation metrics. The sensor setup for data acquisition provides event-based stereo data as well as ground truth in form of depth information. The data acquisition also comprises pre-processing and data fusion. We used ROS–Robot Operating System–as framework to integrate the event cameras, the stereo cameras, the experiment scripts and the communication.

### Data Acquisition

As we require a sensor setup capable of capturing calibrated and synchronized data from event-based sensors and providing a ground truth for the depth information simultaneously, we developed a hardware framework consisting of three sensors. Generating calibrated and synchronized event-based data with ground truth is hindered as operating three cameras simultaneously causes severe technical issues regarding the USB controller and are thus limited by its bandwidth. The problems range from bad performance, complete system crashes, incomplete data recordings to massive time lags. Even though some of the underlying issues are solvable with additional hardware, we found it to be a more stable and accessible approach to record the stereo streams and point cloud separately on different host computers. Consequently, the data acquisition yields four different files, the event stream of each sensor as a raw-file, a rosbag^1^ containing the stereo camera’s point cloud and a text file holding the ROS timestamps for the recording’s start and end. In order to prepare the data for evaluation purposes like analyzation and visualization, the recordings have to be pre-processed and merged.

**FIGURE 1:**
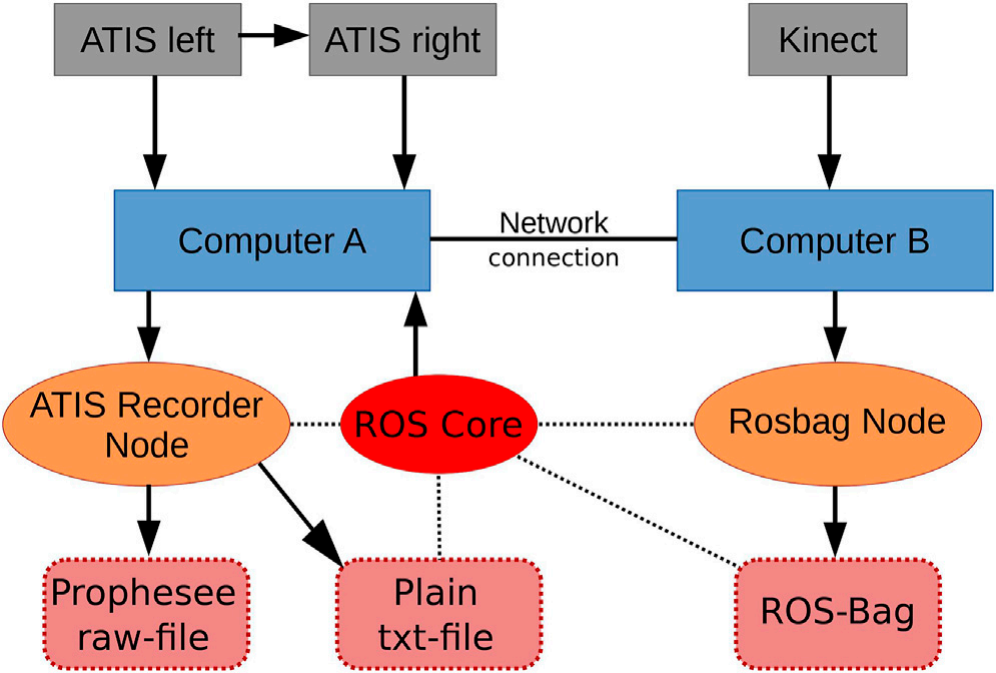
Recording setup consisting of three sensors (gray), two computers (blue), the ROS core (red), the two recorder ROS-nodes (orange) and the different data structures (dark red). The arrows represent direct data transfer while dashed lines symbolize which structures are synchronized via the roscore’s timestamps.

**FIGURE 2:**
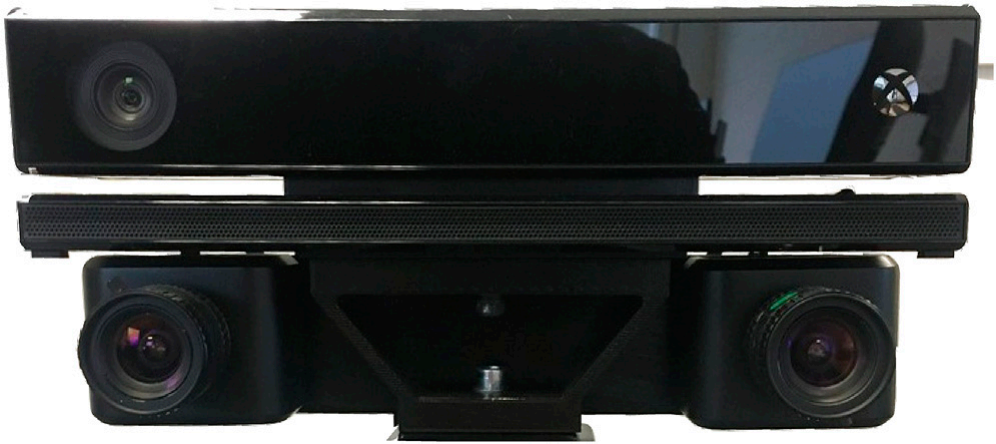
Sensor setup featuring the Kinect mounted on top and two ATIS underneath. All sensors are fixed on a 3D-printed camera mount. The blue, red and gray cables are connecting the trigger IN/OUT pins required for synchronizing the event-based sensor’s individual clocks.

In Figure 1, the developed ROS environment for data acquisition including all hardware and software components as well as their synergies is visualized. For publishing the Kinect’s data, we use IAI_Kinect2^2^ based on libfreenect2 Xiang et al. (2016), while we extended the ROS driver of the ATIS sensor’s manufacturer (Prophesee) to support stereo publishing with synchronized time stamps.^3^ The computers communicate via an Ethernet connection to each other. The ATIS sensors are connected and synchronized using trigger IN-/OUT-Pins enabling the left ATIS to force the right one to synchronize its timestamps. The output streams of both ATIS are recorded by a ROS-Node hosted by Computer A. The stereo camera’s point cloud is recorded by a rosbag-node which is hosted by Computer B. The synchronized timestamp of both recordings is provided by Computer A. A photography of the hardware setup of all three sensors is shown in Figure 2. As datasets recorded this way naturally consist of different formats, the data has to merged. This is done by subtracting the starting timestamp from the plain text file (see Figure 1) from each point cloud’s timestamp and subsequently assign each pair of potentially corresponding events to the point cloud with the closest timestamp.

### Calibration

In order to determine the geometrical relations between the applied cameras, as well as the mapping from the camera’s lenses to their sensors, a calibration is required. This process involves four cameras: two event cameras (*ATIS left* and *ATIS right*), the Kinect’s infrared sensor (IR) and its RGB camera (RGB). Conventionally, a printed checkerboard pattern is used to perform camera calibration. For neuromorphic vision this is not possible, as event-based sensors cannot observe static scenes. Instead, a blinking checkerboard on a monitor is used. However, the IR cannot observe the checkerboard on a screen. To retrieve the geometrical relations of the IR to one of the event cameras, we can calibrate the IR with the RGB using a printed pattern. Subsequently, the RGB is calibrated with one of the event cameras using a monitor. After that we can convert a point from the IR’s to the event camera’s coordinate system by applying chained homogeneous transformation. In order to be able to calibrate the event cameras using a standard calibrator, we need to extract a static checkerboard pattern from the recorded event streams. To do so a scene of 500 ms of the blinking checkerboard is recorded. Afterward the recorded event streams can be accumulated to extract a static image. Subsequently, the accumulation is median filtered, inverted and normalized to gain a 8 bit grayscale image. The images generated this way can be fed to a calibrator like Matlab or OpenCV to extract the intrinsic and extrinsic camera parameters.

Table 1 shows the results of the calibration process. It includes the monocular calibrations for each camera individually, four in total, as well as the three stereo calibrations relating the cameras to each other. It can be seen that for the event camera’s calibrations only a subset (*selections*) of all recordings *#recordings* is used. The reason is that the pattern detection for the event camera is not run online as it is for the stereo camera Xiang et al. (2016) and therefore, the pattern is more prone to detection failures due to blur.

**TABLE 1:** Calibration results. *#recordings* refers to how many images were taken in the calibration process, while *#selections* indicates how many pictures the calibration algorithm was applied to (post outlier deletion). *Error* is the Reprojection error. IR refers to the infrared sensor of the Kinect and RGB to the color camera.

Sensor	#Recordings	#Selections	Error
ATIS left	42	34	0.0837
ATIS right	46	36	0.0984
ATIS stereo	41	34	0.1399
Kinect IR	50	50	0.1028
Kinect RGB	61	61	0.1633
Kinect stereo	55	55	0.1529
Kinect to ATIS	45	30	0.4264

### Metrics

To compare different algorithms in a reproducible manner, we define two quantitative criteria 1) *performance* and 2) *precision*, as stated in Table 2.

**TABLE 2:** The quantitative criteria of *performance* and *precision* defining our evaluation metrics and their sub classes.

Performance	Reconstructed points per second (rps)
Precision	Median error
	# False matches

The first category performance, is an important factor as it limits potential use cases of the algorithm drastically. A low performing algorithm is not suited for real-time processing which is indispensable in areas such as human robot collaboration or autonomous driving. As the performance is strongly influenced by the hardware used for processing, it is crucial to specify the applied system and to compare all algorithms on the same hardware. In neuromorphic vision, many algorithms are heavily dependent on neuromorphic hardware. Comparing their performance to algorithms that do not make use of neuromorphic hardware is extremely challenging. In conventional computer vision performance is often measured in amount of processed frames per second. Hereby, real-time capability can be deduced easily. If the amount of processed frames per second exceeds the camera’s frame rate, the algorithm is capable of real-time processing. In contrast to that, neuromorphic sensors have no frame rate and the amount of events generated heavily depends on the scene and illumination. Consequently, we used the amount of reconstructed points per second (rps) as a valid and meaningful value. The second category is about the precision of the 3D reconstruction. As a ground truth to evaluate against, we use a point cloud. To compare it with the reconstruction values, those must be transformed to the stereo camera’s coordinate system. Afterward, each point from the reconstructed point cloud is matched with the closest point of the stereo camera in a spatio-temporal space. We can now define the minimum distance $d_{\min}$ of a reconstructed point p to the stereo camera’s point cloud K as

$$d_{\min}\left(p\right)=\min_{K}\left(\left||p-k|\right|\right),k\in K$$ (1)

Applying Equationfootnote:footnote 1, the precision of the algorithm can be specified by two criterions, the *median error* and the number of *false matches*. Regarding the mean error

$$m_{prec,1}=median\left(d_{\min}\right)$$ (2)

the error is calculated as the distance of the matched point pairs. As falsely matched events can cause extremely high errors for single points, influencing the mean value overproportionally, the mean value is not meaningful in this case. The median, however, is not influenced by such gross outliers which makes it the better metric for this use case. As the second criterion (*false matches*), we define all points whose distance to their corresponding ground truth point is greater than 10% of their depth value. The threshold is relative to the depth because the Kinect’s depth accuracy deteriorates with distance. The number of false matches can then be used to calculate the algorithm’s false *match ratio* similar to Ieng et al. (2018). It is defined as the number of false matches divided by the number of reconstructed points

$$m_{prec,2}=\left(\frac{{\sum^{\text{​}}}_{i=0}^{N}H\left(d_{\min,i}-0.1\cdot z_{i}\right)}{N}\right),$$ (3)

with H(x) being the Heaviside function, *N* being the number of points and $z_{i}$ being the depth of the *i* ^th^ point.

Regarding the applicability of an algorithm, two characteristics of event-based stereo vision techniques are crucial. Firstly, the ability to process asynchronous event streams in a performant way and secondly, the applicability to neuromorphic hardware. In case an algorithm cannot process event streams asynchronously, it requires frame reconstruction. Hence, event-buffering is often necessary which slows down the system dramatically. On top of this, such an algorithm does not profit from the high temporal resolution of event-based sensors. Regarding the second characteristic, the applicability to neuromorphic hardware has benefits in terms of computation times making it an important feature for real-time applications. In order to qualify for that, algorithms must operate parallelized and asynchronously. A property that usually only applies to SNN-based approaches. Even though a method’s applicability is very expressive and might already limit the algorithm’s use cases, it is complicated to judge and to use for comparison. Moreover, the applicability is implicitly already covered by the category *Performance*. Therefor it is not considered as a separate criterion.

### Potential Applications and Guidelines

To enable reproducibility, key points for potential applications are provided. Even though, these constraints might reduce the number of potential application areas we believe it adds value to specify ideal applications. Regarding the topic area we focus on a static camera setup and a moving scene. Hence, areas of interest include industrial robotics and surveillance. Tasks like autonomous driving and especially drones are less suitable. Both are also inappropriate because of the preferred distance from the sensor to the recorded objects. The principle of stereo vision is generally limited in distance, because disparities can only be achieved in pixel positions, which are naturally discrete. An object at infinity will always yield in a disparity of zero assuming a finite distance of the cameras. For parallel images the depth *z* of a point can be calculated as (z=f⋅b/d⋅p) with camera’s focal length *f*, the distance between the cameras *b*, the disparity *d* and the sensor’s pixel pitch *p*. Assuming the ATIS parameters and a distance between the cameras of 20 cm, an object in a distance of 80°m would yield a disparity of one. For objects further away a disparity can not be perceived therefor the maximum distance is 80°m. Very close points can generally not be observed by both cameras. However, the view angle and the distance between both cameras are additional limiting factors for the minimum distance. The overlap of the camera’s fields of view grow with the objects’ distance to the setup. The closest point observed by both sensors is at d=tan(π−α/2)⋅(b/2) with alpha being the lens’s angle of view and *b* the distance between both sensors. Assuming the ATIS’s horizontal angle of view of 56.3 and a distance of 20 cm this yields to approximately 18.7 cm. An overlap of the two camera’s field of view of 80% is thus achieved at a distance of approximately 1.68°m.

Regarding guidelines for future users we suggest the following practical steps:

1. Sensor setup: Build a setup of two event-based sensors and one depth camera. For example two ATIS and one Kinect sensor mounted on a 3D printed fixture and a tripod.
2. Calibration:
  - Mono calibration for all four sensors
  - stereo calibration ATIS to ATIS
  - stereo calibration of one ATIS to the RGB sensor
  - stereo calibration of the RGB sensor to the IR sensor
3. Calculate the projection matrix ATIS to IR by using the projections from ATIS to RGB and RGB to IR.
4. Record evaluation scenarios with synchronized timestamps. Synchronization can be achieved with the proposed data acquisition method (see *Data Acquisition*).
5. For each algorithm to be evaluated:
  - Calculate the 3D reconstruction of the scene
  - Match every reconstructed point to the closest point of the Kinect’s point cloud with the respective timestamp.
6. Calculate the distances.
7. Calculate the proposed criteria.

## Experiments

The event-based sensor we used to implement and evaluate our concept is Prophesee’s^4^ Evaluation Kit Gen3 HVGA-EM. The sensor is an implementation of the ATIS architecture introduced in Posch et al. (2011). Important specifications of the ATIS regarding our benchmark environment are listed in Table 3.^5^

**TABLE 3:** Specifications of the Evaluation Kit Gen3 HVGA-EM sensor used for this work.

Resolution	480 × 360
Pixel pitch	20 μm
Optical format	3/4 inch
Latency	200 μs
Temporal resolution	1 μs
Field of view (H × V)	56.3 × 43.7 °
Dynamic range	120 dB
Interface	USB 3.0

The two depth cameras that were considered for this work are the *Kinect v2* and the *RealSense D435*. Even though the RealSense has a higher frame rate, resolution and a larger Field of View, many publications in the field of depth reconstruction on event-based data used the Kinect as the source of ground truth Haessig et al. (2019); Ieng et al. (2018); Osswald et al. (2017). As this sensor supports a reconstruction precision of a few mm at 50 and 3 cm at 3 m Khoshelham (2012) and MacKnojia et al. (2012) it proved to be sufficiently for our use case.

### Test Candidate

**FIGURE 3:**
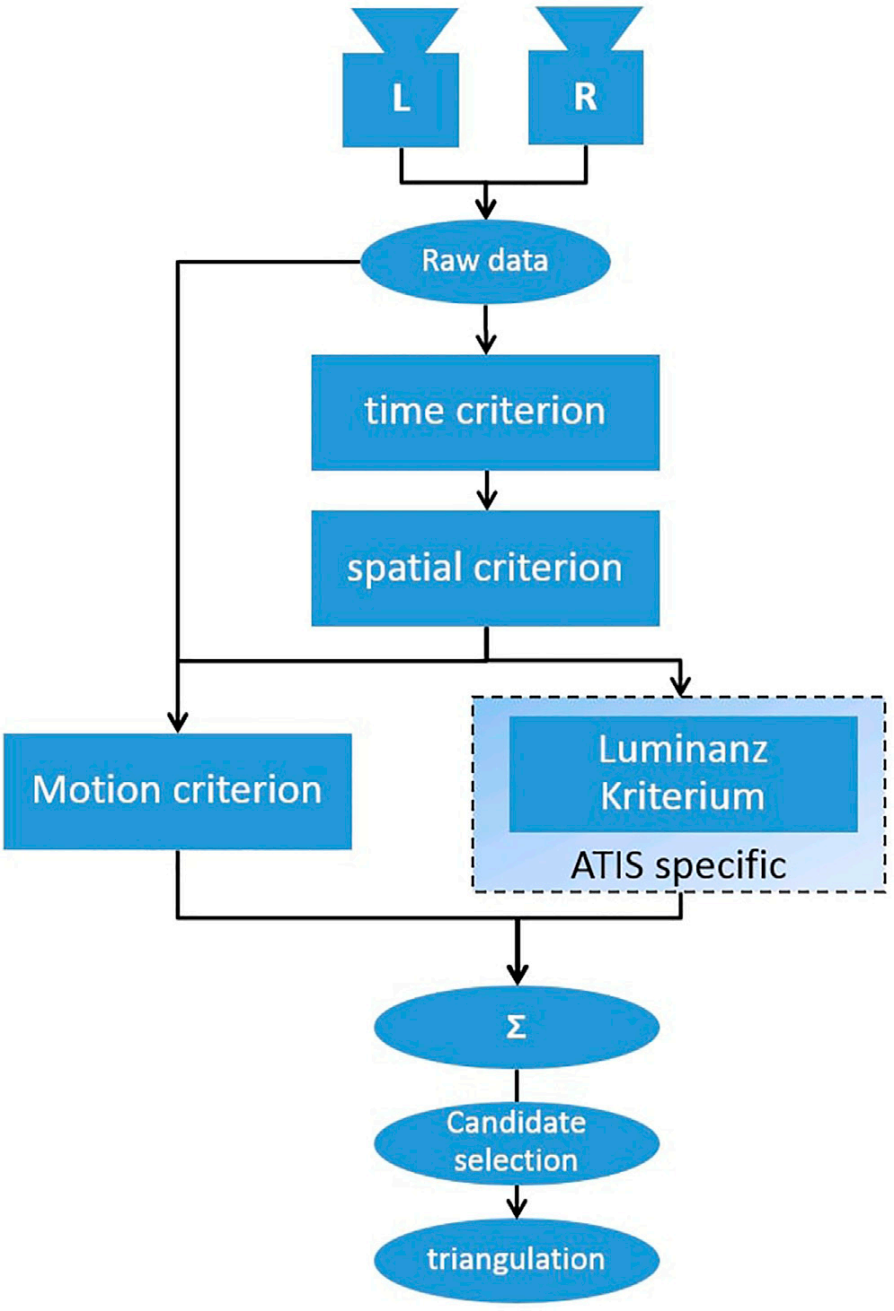
High-level flow chart of the evaluated algorithm. Correspondences are determined by four criterions; the time criterion comparing event’s time stamps, the spatial criterion exploiting epipolar geometry, luminance criterion comparing luminance values and the motion criterion calculating motion fields.

To allow an evaluation of our environment, a stereo matching algorithm for event streams Ieng et al. (2018) is chosen and implemented. It is applied to recorded event streams of different experiments. The reconstruction results are evaluated by comparison with the ground truth. The implemented algorithm is shown in Figure 3. It calculates the probability of two events to be triggered by the same real-world point, by applying four criteria in the form of energy functionals and then minimizing their sum. The initial event pairs are chosen by applying two tolerance thresholds: A temporal tolerance, which says that events can appear no longer than $\varepsilon_{T}$ apart from each other and the a spatial tolerance, which says that events cannot violate the epipolar constraints by more than $\varepsilon_{S}$ pixels. For each pair of events that meets these two conditions, the four criteria are evaluated. These are the temporal, spatial, motion and luminance criteria. The energy functional for the temporal criteria equals zero if the events timestamps match and one if the timestamp’s difference is equal to the temporal tolerance. The energy functional for the spatial criteria can be calculated analogously using the spatial tolerance. The motion criteria defines a motion surface consisting of past events in the neighborhood. The more similar these motion surfaces of the two events are, the lower is the value of the energy functional. The last criteria, the luminance, measures the similarity in gray value change triggered by the events. This criteria was not implemented here. However, the authors of Ieng et al. (2018) report very similar matching precision applying just three or all four criteria.

### Evaluation Scenarios

**FIGURE 4:**
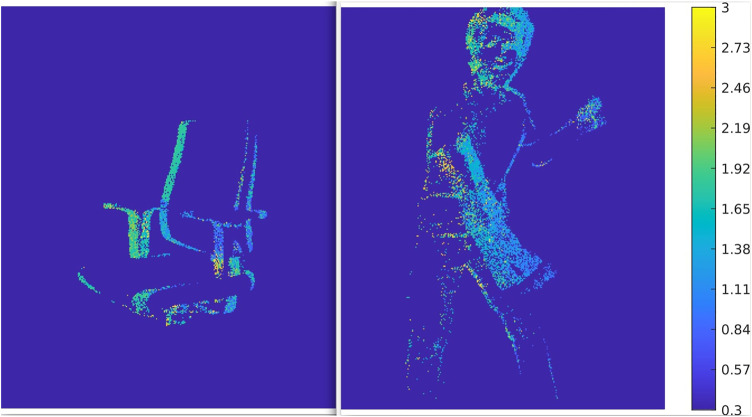
Depth maps of reconstructed scenes from synchronized event-streams of two ATIS. The color bar on the side states which color represents which distance. It can be seen that the areas closest to the camera like the left armrest or the left arm, are of a darker blue as these object’s distance to the sensor about 0.3–1.0 m. Respectively, areas further away, like the right edge of the back rest or the head, are colored green, representing a distance of ca. 1.6–2.1 m.

In conventional computer vision, a stereo algorithm would first be evaluated using very easy scenes including only a few objects and a completely static scene. Afterward, dynamics and more complexity would be added to the scene. Thereby, algorithms could be evaluated for their behavior with static and dynamic scenes separately, as it is done in Giancola et al. (2018). For event-based vision, this is not possible, because event-based sensors cannot record static scenes. Hence, the most important criteria for a test scenario in this benchmark environment is that the scenario includes one or multiple moving objects. Despite that, an object should be sufficiently big to cover a great part of the field of view of the camera setup. Also, the object should be placed centered in a distance of approximately 1–3 m as this is the optimal position for highest accuracy of the Kinect Yang et al. (2015). For the experiments of this work, two different scenarios are used. One scenario shows a spinning office chair and the other one a moving man. Both scenarios have been arranged as described above. As an example, reconstructed depth maps of those scenarios are shown in Figure 4.

## Results

After recording, prerocessing and merging the event-based and depth data for analysis, as described in *Data Acquisition*, we evaluate the quality of reconstructions in this section. This is done using the metrics defined in *Metrics*. As mentioned in *Metrics* two properties of the algorithm are decisive; its ability to efficiently process asynchronous data and its scalability to neuromorphic hardware. Regarding the former, the method presented in Ieng et al. (2018) processes the asynchronous input in a semi-asynchronous fashion. Event pairs are matched within a certain time window. Hence, events need to be buffered for the length of this window. This is different to SNN-based techniques which instantly process every event without any delay. However, it does not require to buffer a whole frame and is–at least from this perspective–superior to area- and feature-based approaches based on conventional 3D reconstruction. The time windows required for buffering may be set manually. In the scope of this work, a time window of 100 μs is used. Hence, the buffering is shorter than a millisecond and does not noticeably influence the temporal resolution of the output stream. The algorithm is not an SNN-based approach. It can therefore not be applied to neuromorphic hardware. While this can be a disadvantage when compared to an algorithm that is executed on neuromorphic hardware, it can also be seen as an advantage: The algorithm is sufficiently efficient without the need for dedicated hardware. This enhances its accessibility and lowers the barriers to experiment with it. As suggested in Table 2, an analysis of its performance is carried out in *Performance Analysis*. This is complemented with an evaluation regarding the algorithm’s precision, considering the median error and the number of false matches in *Precision Analysis*.

### Performance Analysis

As the performance of an algorithm is highly depending on the applied hardware, the key specifications of the computer all experiments were executed on is listed in Table 4.

**TABLE 4:** Specifications of the executing computer.

Operating system	Ubuntu 18.04.4 LTS
Processor	Intel Core i7-7700HQ 2.7 GHz
Memory	32 GB DDR4-2400

**FIGURE 5:**
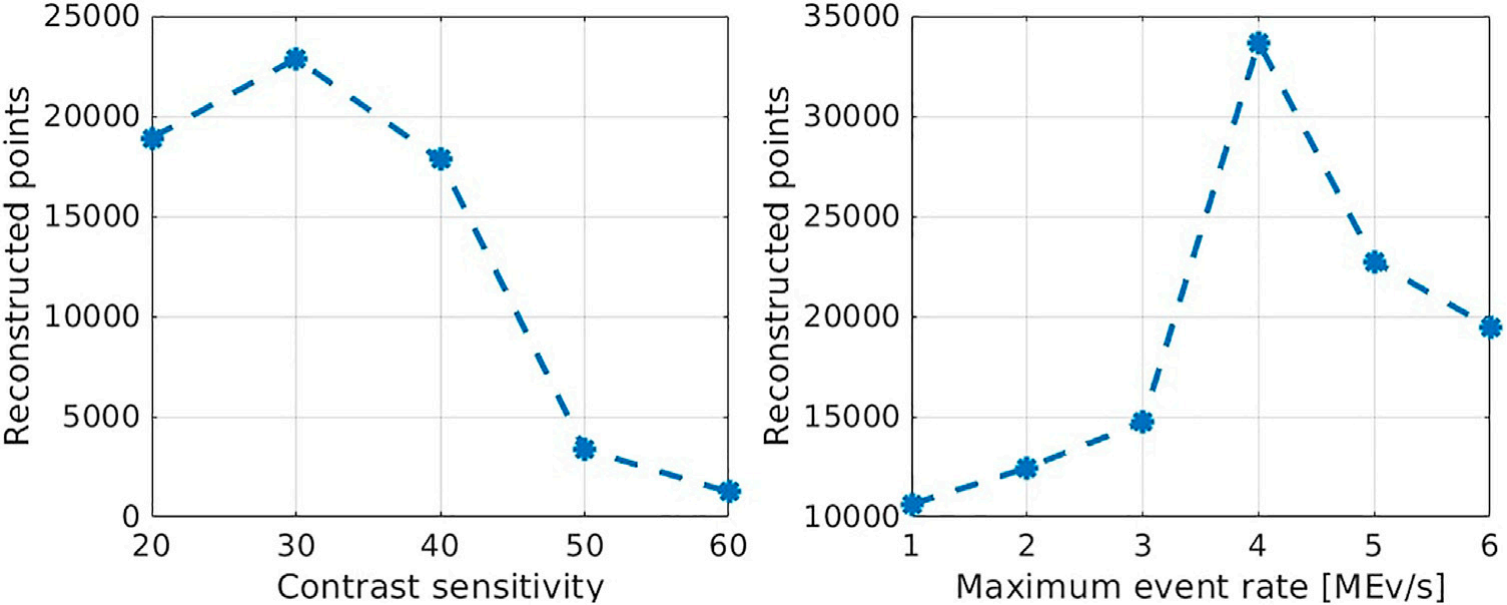
Number of reconstructed points in different experiments with different camera settings. On the left, the experiments have been carried out with a maximum event rate of 5 MEv/s and varying contrast sensitivities. On the right, a fixed contrast sensitivity of 30 and varying maximum event rates were used. For each data point represents the mean value of five trials.

The key parameter utilized for the performance analysis is the number of reconstructed points per second (rps). This number can vary from execution to execution, which decreases its expressiveness. To counteract this, the algorithm is applied five times on the same data and the mean value of rps is used. The results of the performance evaluation are shown in Figure 5.

The average reconstruction rate is almost 35 k rps. Although, it is noteworthy that this result is highly affected by the camera parameters. Without averaging about 42 k rps where reached for single experiments. The authors of Ieng et al. (2018) claim to achieve rates of 47 k rps to 49 k rps. However, they do not provide details under what conditions these rates were determined. As can be seen in Figure 5 the algorithm performs very poorly for high contrast sensitivities. This behavior is due to the algorithm’s runtime complexity being quadratic to the temporal density of the events. Another weak spot is exposed for low maximum event rates of up to 3 MEvs. Unfortunately, the cause for this is not entirely clear. A possible explanation is that high dead times of pixels lead to a high number of events without a possible matching candidate in their spatiotemporal window. Those events obviously do not contribute to the reconstruction. Regarding the influence of the camera parameters the results of the performance analysis are consistent with the comparison to the point clouds. It can be concluded that the contrast sensitivity should not be set higher than 40 and the maximum event rate should be at least 4 MEv/s.

### Precision Analysis

To evaluate the reconstruction results of the event-based stereo matching algorithm in a meaningful manner, they are compared to the ground truth generated by the stereo camera. Hereby, the term *distance* always refers to the distance of a reconstructed point to its closest point in the corresponding point cloud.

**FIGURE 6:**
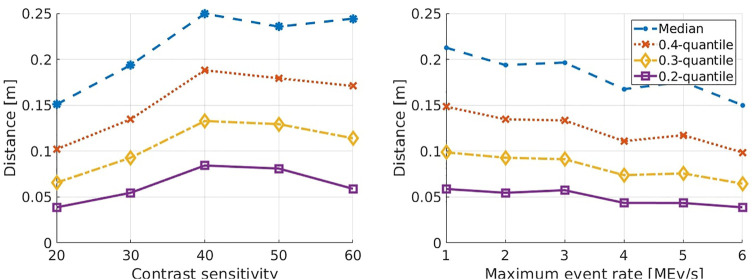
The graphs show how certain camera parameters may influence the measured distances between the reconstructed points and the closest once of the point cloud. The distances are plotted for different contrast sensitivities on the left and for over the maximum event rate on the right. Each plot shows four lines for the median at 0.4, 0.3 and 0.2 quantiles respectively.

Figure 6 displays the median distance of all points for multiple experiments. With a range from 15 cm up to 25 cm the median distances are relatively big. This plot also reveals how the camera parameters influence the accuracy of reconstructions. For instance, higher contrast sensitivities cause a larger median error because higher contrast sensitivities yield higher event rates and a more noisy event stream. Hence, the algorithm generates more false matches for events triggered by reflections for instance. This effect is lessened for smaller quantiles as the sensitivity does not affect the triangulation and thereby the accuracy. For the maximum event rate, the opposite effect can be observed. The higher the parameter, the lower the distances. We assume that high pixel dead times often prevent the generation of events that otherwise could be matched correctly. The relatively large median distance in Figure 6 is caused by three different problems. The first one is, that the algorithm in Ieng et al. (2018) produces a high rate of mismatches. The authors estimated a quote of false matches of 6̃0%. Assuming this quote, the curves for the 0.4-quantile show that the maximum distance of the correctly matched points is between 10–15 cm depending on the camera parameters. The second problem is the relatively low resolution and at the same time comparatively big pixels of the ATIS. Bigger pixels introduce a higher uncertainty in the triangulation process as the 3D space that is projected to a single pixel is greater than with a smaller pixel size. The root of the third problem is the calibration process which introduces an uncertainty since the ATIS is not directly calibrated with the Kinect’s IR sensor. Hence, the coordinate transformation of the reconstructed points to the IR’s coordinate system also has a higher error.

**FIGURE 7:**
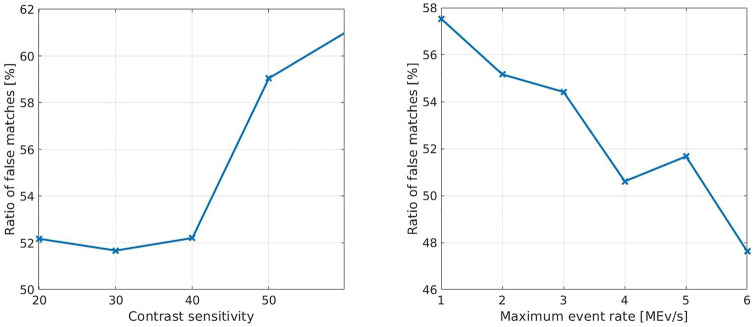
Relative representation of false matches over several experiments with different camera parameters. How contrast sensitivity affects the algorithm’s results is shown on the left and how the results relate to the maximum event rate is shown on the right.

In Figure 7, the ratio of false matches is displayed. During this experiment, different tests with different camera parameters were compared. As described in *Metrics*, a reconstructed point is considered a false match when its distance to the point cloud is greater than 10% of the depth value of the corresponding Kinect point.

**FIGURE 8:**
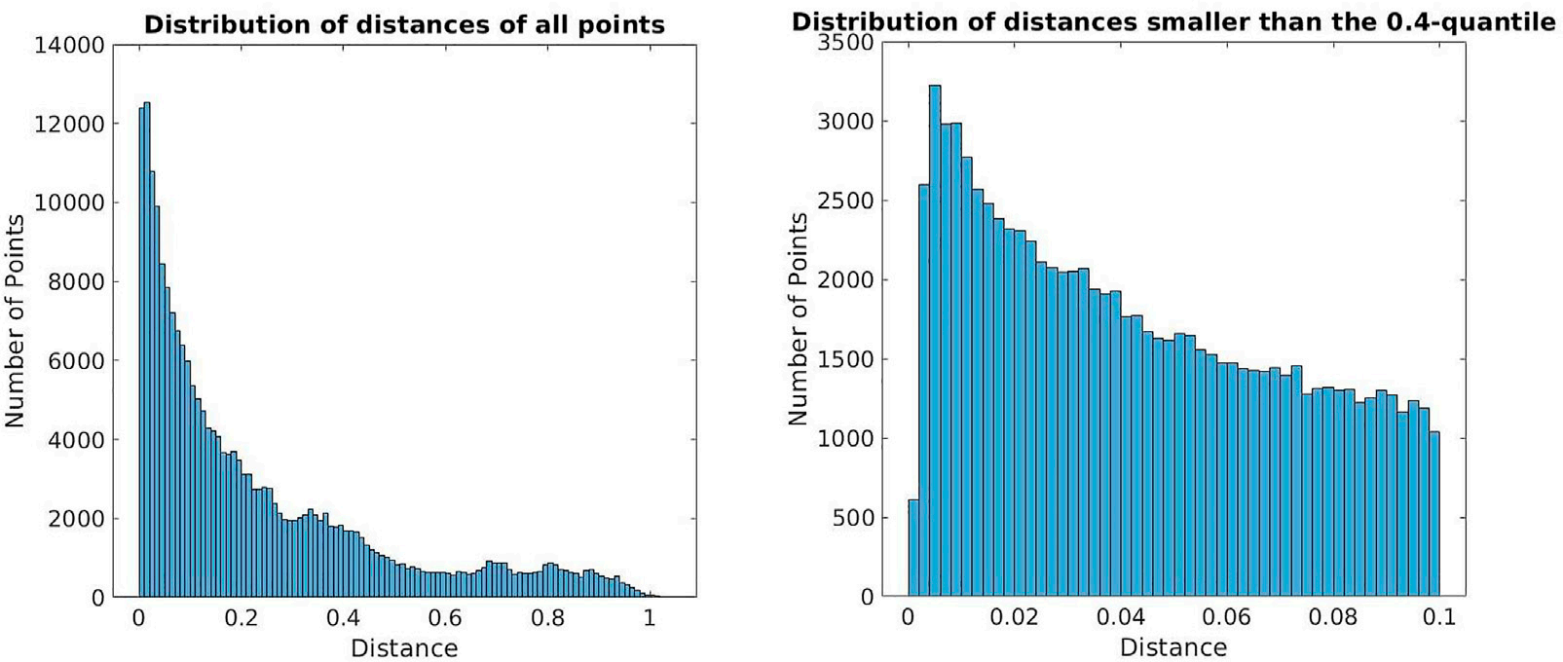
Distribution of distances between the reconstructed points and the ground truth. On the left all reconstructed points are considered while the right graph takes only the points below the 0.4-quantile into account.

The distribution of the distances between the reconstructed points from the stereo event streams and the point clouds is shown in Figure 8. The graphs show the number of points that could be reconstructed in different experiments with different camera settings. On the left the experiments have been carried out with a maximum event rate of 5 MEv/s and varying contrast sensitivities. On the right experiments were carried out with a fixed contrast sensitivity of 30 and varying maximum event rates. Hence, the left part shows all reconstructed points and the right one only relatively good matches, which do not exceed distances of 10 cm. Only about 40% of the reconstructed points fall into this category. For each data point in the graphs the algorithm was applied five times and the mean value of reconstructed points for the five executions was used. In conclusion, Figure 8 illustrates well that the performance depends heavily on the camera settings.

## Conclusions and Future Works

Safe human machine collaboration is one of the big challenges in automation and robotics. Neuromorphic vision systems inducing low latency, a very high dynamic range, and no motion blur, are one possible solution. However, due to their fundamentally different output form, event streams instead of intensity images, conventional computer vision provides no adequate processing techniques for them. Therefore, research exploiting the sensor’s asynchronous principle of operation and thereby derived high temporal resolution is very active. Many new approaches for depth reconstruction from event-based data have been presented. This work provides a robust and flexible way to evaluate this multitude of new algorithms by introducing a comprehensive benchmark environment for depth reconstruction. It covers all aspects necessary to evaluate 3D reconstruction algorithms on event-based data. Therefore, we presented all required techniques for data acquisition and calibration. The theoretical contribution was complemented with a metric to improve comparability. To test our approach, we implemented an evaluation candidate and realized some evaluation scenarios. These experiments have been evaluated regarding the previously introduced metrics. The distributed architecture we designed works well and yields a robust and reliable synchronization of all applied sensors, as could be proven by the evaluation. Despite its distributed design, the setup provides an easy-to-use interface for recording, making the environment very flexible. An interesting follow-up project would be to utilize this environment to create a dataset similar to Zhu et al. (2018); Binas et al. (2017). The datasets are focused on automotive applications and use the DAVIS. To the best of our knowledge, there is no data set providing stereo event streams of an ATIS sensor with a ground truth. Publishing a data set with a different sensor and different application area like industrial robotics could greatly benefit the research community in the field. The ATIS is a relatively sophisticated neuromorphic sensor regarding pixel size, resolution and dynamic range. However, as particular attention is paid to neuromorphic systems lately, more powerful sensors have been brought onto the market. To improve an evaluation for event-based stereo vision methods, it would be interesting to apply our benchmark environment with more potent technologies. Beyond that, an good future project would be to integrate other stereo vision algorithms for evaluation using this environment. The variety of approaches is large and many of them seem promising. Implementing the approaches is, however, very timeconsuming and open source implementations of the authors themselves have not been published in most cases.

## Data Availability

The raw data supporting the conclusions of this article will be made available by the authors, without undue reservation.

## Footnotes

1 A ROS tool for recording and playing data. For more information see http://wiki.ros.org/rosbag

2 Published in https://github.com/code-iai/iai\_kinect2

3
https://github.com/prophesee-ai/prophesee_ros_stereo_driver/pull/1

4
https://www.prophesee.ai/

5 Data source: https://support.prophesee.ai/portal/kb/articles/hvga-cd-em-event-based-evaluation-kit
